# *Aspidosperma pyrifolium* Has Anti-Inflammatory Properties: An Experimental Study in Mice with Peritonitis Induced by *Tityus serrulatus* Venom or Carrageenan

**DOI:** 10.3390/ijms18112248

**Published:** 2017-10-26

**Authors:** Maíra Conceição Jerônimo de Souza Lima, Mariana Angélica Oliveira Bitencourt, Allanny Alves Furtado, Manoela Torres-Rêgo, Emerson Michell da Silva Siqueira, Ruth Medeiros Oliveira, Hugo Alexandre Oliveira Rocha, Keyla Borges Ferreira Rocha, Arnóbio Antônio da Silva-Júnior, Silvana Maria Zucolotto, Matheus de Freitas Fernandes-Pedrosa

**Affiliations:** 1 Laboratory of Technology and Pharmaceutical Biotechnology (Tecbiofar), Department of Pharmaceutical Sciences, Faculty of Pharmacy, Federal University of Rio Grande do Norte, Rua General Gustavo Cordeiro de Farias, S/N, Petrópolis 59012-570, Natal, Brazil; mairalima4@hotmail.com (M.C.J.d.S.L.); mariana.bitencourt@unp.br (M.A.O.B.); allannyfurtado@hotmail.com (A.A.F.); manoelatorres_rn@hotmail.com (M.T.-R.); arnobiosilva@gmail.com (A.A.d.S.-J.); 2 Laboratory of Pharmacognosy (PNBio), Department of Pharmaceutical Sciences, Faculty of Pharmacy, Federal University of Rio Grande do Norte, Rua General Gustavo Cordeiro de Farias, S/N, Petrópolis 59012-570, Natal, Brazil; siqueira_emerson@hotmail.com (E.M.d.S.S.); szucolotto@hotmail.com (S.M.Z.); 3 Laboratory of Biotechnology of Natural Biopolymers, Department of Biochemistry, Bioscience Center, Campus Universitário, Federal University of Rio Grande do Norte, Avenida Senador Salgado Filho, 3000, Lagoa Nova 59072-970, Natal, Brazil; rmo_85@hotmail.com (R.M.O.); hugo-alexandre@uol.com.br (H.A.O.R.); 4 Laboratory of Pathology, Departament of Pathology, Federal University of Rio Grande do Norte, Rua General Gustavo Cordeiro de Farias, S/N, Petrópolis 59012-570, Natal, Brazil; keyla.rocha@uol.com.br

**Keywords:** anti-inflammatory activity, *Aspidosperma pyrifolium*, carrageenan, rutin, scorpion venom, *Tityus serrulatus*

## Abstract

Scorpions of the genus *Tityus* are responsible for the majority of envenomation in Brazil, the *Tityus serrulatus* species being the most common and dangerous in South America. In this approach, we have investigated the ability of the aqueous extract from the leaves of *Aspidosperma pyrifolium* in reducing carrageenan-induced inflammation and the inflammation induced by *T. serrulatus* envenomation in mice. We also evaluated the cytotoxic effects of this extract, using the 3-(4,5-dimethythiazol-2-yl)-2,5-diphenyl-2H-tetrazolium (MTT) assay and the results revealed that the extract is safe. Analysis by High Performance Liquid Chromatography coupled with Diode Array Detector (HPLC-DAD) and Liquid Chromatography Coupled with Mass Spectrometry with Diode Array Detection (LC-DAD-MS) showed one major chemical component, the flavonoid rutin and phenolics compounds. For in vivo studies in carrageenan-induced peritonitis model, mice received extracts, dexamethasone, rutin or saline, before administration of carrageenan. For venom-induced inflammation model, animals received *T. serrulatus* venom and were, simultaneously, treated with extracts, antivenom, rutin or saline. The extract and rutin showed a reduction in the cell migration into the peritoneal cavity, and in the same way the envenomated animals also showed reduction of edema, inflammatory cell infiltration and vasodilation in lungs. This is an original study revealing the potential action of *A. pyrifolium* against inflammation caused by *Tityus serrulatus* venom and carrageenan, revealing that this extract and its bioactive molecules, specifically rutin, may present potential anti-inflammatory application.

## 1. Introduction

The scorpion genus *Tityus* is responsible for the majority of scorpion envenomation in Brazil, and, in particular, the species *Tityus serrulatus* (Buthidae) is the most common and harmful. In South America, this species is the most dangerous due to the high toxicity of its venom. The envenomation induced by scorpions of the genus *Tityus* spp. is a more serious concern for children and the elderly than adults [1,2,3,4]. 

*T. serrulatus* venom (VTs) causes pulmonary edema, resulting in an increase in lung permeability by vasoactive substances released by the inflammatory process. The pathogenesis of this edema is very complex, therefore, envenomation by this species can be severe, and deaths are often caused by acute pulmonary edema [5,6]. Others symptoms displayed by victims of scorpion accidents are fever, restlessness, excessive salivation, lacrimation, increased gastrointestinal motility, respiratory, cardiac arrhythmias, acute pulmonary inflammation, hypertension followed by hypotension, heart failure, and cardiogenic shock. In part, these clinical manifestations may be observed by the presence of neurotoxic components in the venom which interact with sodium and potassium channels in nerve endings [7,8]. 

Antivenom therapy is the treatment of choice for severe cases of envenomation by scorpion [9]. The administration of antivenom should occur as soon as possible so there is the neutralization of systemic effects caused by envenomation. Not all victims have access to this kind of treatment because they live in conditions where medical help is not readily available. Some studies suggest that it is possible to use a plant extract or an herbal drug as an alternative treatment for envenomation by venomous animals, in an attempt to minimize the effects of local inflammatory poison [10,11,12]. Recent studies by our group have demonstrated the beneficial effect of the use of plant extracts in the local treatment of snakebite [13], but there are few studies exhibiting the effectiveness of such treatment in envenomation induced by scorpion sting [10,14,15,16].

The Apocynaceae family has considerable economic and medicinal importance [17,18]. *Aspidosperma pyrifolium*, popularly known as “pereiro”, mainly grows in the semi-arid northeastern region of Brazil [19], and has several applications in folk medicine, such as relief of benign prostatic hyperplasia, erectile dysfunction and inflammation [18,20]. Popular reports in farms in the State of Rio Grande do Norte, Brazil, described that the old-aged-people used the species for the treatment of dizziness, urinary disorders, wound healing and inflammation.

On Asclepiadoideae plants, which are a subfamily of Apocynaceae, phytochemical studies verified the presence of phenolic compounds, as well as the flavonoids quercetin, rutin and isorhamnetin [21,22]. In the current study, mainly the presence of flavonoids and phenolic acids were investigated. The flavonoid rutin occurs naturally in various plant species and is a potent antioxidant with a wide spectrum of applications and has been a subject of interest due to its various pharmacological activities, in particular its anti-inflammatory activity [23,24,25,26,27,28]. There are so far no studies focusing on *A. pyrifolium* pharmacological applications against inflammation and envenomation by venomous animals.

The aim of this approach was to evaluate in vivo the ability of the aqueous extract of *A. pyrifolium* and rutin to reduce the carrageenan-induced inflammation and the inflammation induced by *Tityus serrulatus* envenomation in murine model of peritonitis.

## 2. Results

### 2.1. High Performance Liquid Chromatography Coupled with Diode Array Detector (HPLC-DAD)

**Figure 1:**
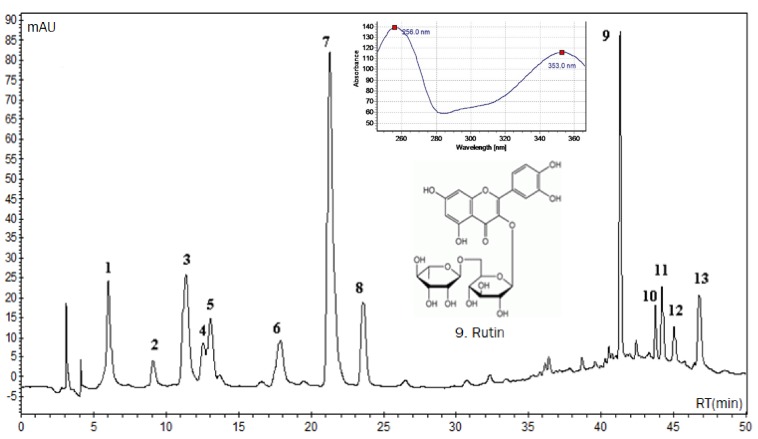
High Performance Liquid Chromatography coupled with Diode Array Detector (HPLC-DAD) chromatogram of aqueous extract from the leaves of *Aspidosperma pyrifolium* (3 mg/mL). The analysis shows one peak (retention time: 41.18 min) corresponding to rutin (inset shows structure and maximum UV absorption at 256 nm and 353 nm). The separation was performed using a Reverse Phase Luna C-18 column (4.6 mm × 250 mm, 5 μm; Phenomenex). The solvent system used was a gradient of 0.3% acetic acid in water: acetonitrile with a flow rate kept at 1.0 mL min^−1^. Detection at 340 nm.

The presence of the flavonoid rutin was confirmed by HPLC-DAD analysis (Figure 1, peak 9) by comparison of the retention time of 41.18 min, UV spectra (256 and 353 nm) and by increasing the area under the peak observed by co-injection of extract and reference standard. Analysis by HPLC-DAD showed that the aqueous extract of *A. pyrifolium* leaves presents a large amount of flavonoid and phenolic derivatives. The UV spectra of peaks unidentified in the chromatogram (UV 340 nm) suggest the presence of phenolic acids (before 25 min) and flavonoids (after the peak of rutin at 41 min).

### 2.2. Liquid Chromatography Coupled with Mass Spectrometry with Diode Array Detection Analysis (LC-DAD-MS)

LC-DAD-MS analyses were performed to determine the molar mass of the compounds under investigation conducted by qualitative liquid chromatography profile. The mass analyzer used was electrospray ionization (ESI-MS) at negative and positive mode. The Mass bank database (Available online: http://www.massbank.jp) was used for to equate the substances spectra. Beyond the rutin suggested by HPLC-DAD analyses, the structure of other seven compounds was identified according to LC-DAD-MS data analysis. 

The spectrum of compound **3** was recorded at *m*/*z* 355 (positive mode), equivalent to the chlorogenic acid (354 u), with a fragment ion at *m*/*z* 163 of caffeic acid portion coming of elimination of quinic acid (192 u) moiety. A respective negative molecular ion [M − H]^−^ was observed at *m*/*z* 353. Compounds **6**, **7** and **8** correspond to chlorogenic acid isomers, *n*-chlogenic, crypto-chlorogenic and iso-chlorogenic acid, respectively. Both exhibit similar mass spectrum but disagree with the retention time [29]. The compound **9** was identified as rutin (610 u), it presented a peak at *m*/*z* 611 of protonated molecule, a fragment at *m*/*z* 465 corresponding to the loss of the rhamnose sugar, and an ion at *m*/*z* 303 corresponding to the quercetin nucleus. For compound **11**, suggested as isorhamnetin-3-*O*-rutinoside (624 u), the [M + H]^+^ ion was observed at *m*/*z* 625 and a protonated aglycone ion [M + H − 308]^+^ at *m*/*z* 317, corresponding to the loss of the rutinose moiety. A respective deprotonated molecular ion was observed at *m*/*z* 623. Compound **12**, the [M + H]^−^ ion in the ESI-MS spectrum, was observed at *m*/*z* 515, which corresponds to the di-*O*-caffeoylquinic acid (516 u), while fragment ion at *m*/*z* 161 was attributed to [M – H − 354]^−^, with the loss of 354 u corresponding to chlorogenic acid moiety elimination and a fragment ion at *m*/*z* 353 attributed to [M − H − 162]^−^, with the loss of 162 u corresponding to caffeic acid. Compound **13**, the [M + H]^+^ ion in the ESI-MS spectrum, was observed at *m*/*z* 517, which corresponds to a isomer of the di-*O*-caffeoylquinic acid (516 u), while fragment ion at *m*/*z* 163 was attributed to [M + H − 354]^+^, with the loss of 354 u corresponding to chlorogenic acid moiety elimination. In negative mode was observed the molecular ion [M − H]^−^ at *m*/*z* 515, a fragment ion at *m*/*z* 353 attributed to [M – H − 162]^−^, with the loss of 162 u corresponding to caffeic acid and a fragment at *m*/*z* 161 [M – H − 354]^−^ corresponding to chlorogenic acid moiety elimination [30]. In isomers compounds **12** and **13***,* it is not possible to identify the position of caffeic acid residues.

### 2.3. Cell Viability

**Figure 2:**
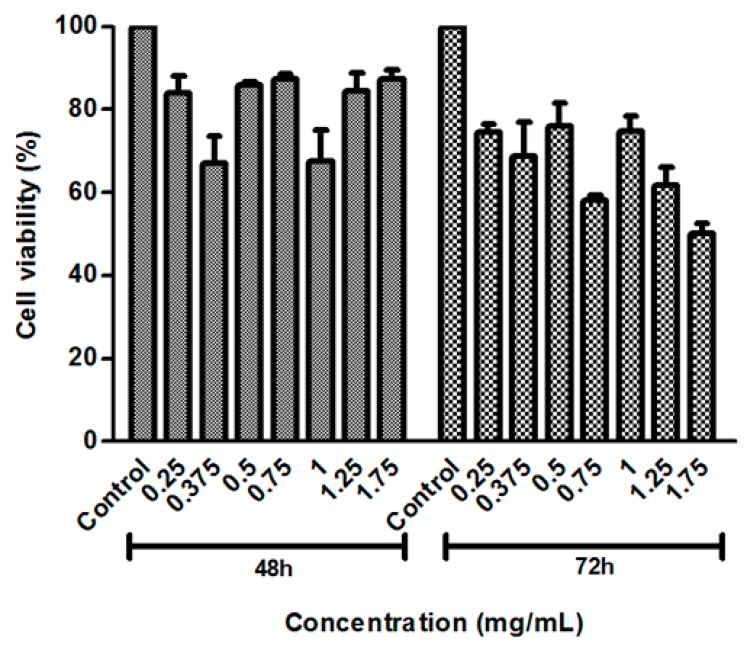
Effects of aqueous extracts from the leaves of *Aspidosperma pyrifolium* on 3T3 cell lines at concentrations from 0.25 to 1.75 mg/mL at 48 and 72 h.

The cell viability of the 3T3 cells was analyzed after 48 and 72 h of incubation with aqueous extract of *A. pyrifolium*, at concentrations between 0.25 and 1.75 mg/mL (Figure 2). The degree of viability was high for all concentrations tested, except for concentration of the 1.75 mg/mL, after 72 h.

### 2.4. Evaluation of Rutin and Aqueous Extract of the Leaves of Aspidosperma pyrifolium in Carrageenan-Induced Peritonitis Model

**Figure 3:**
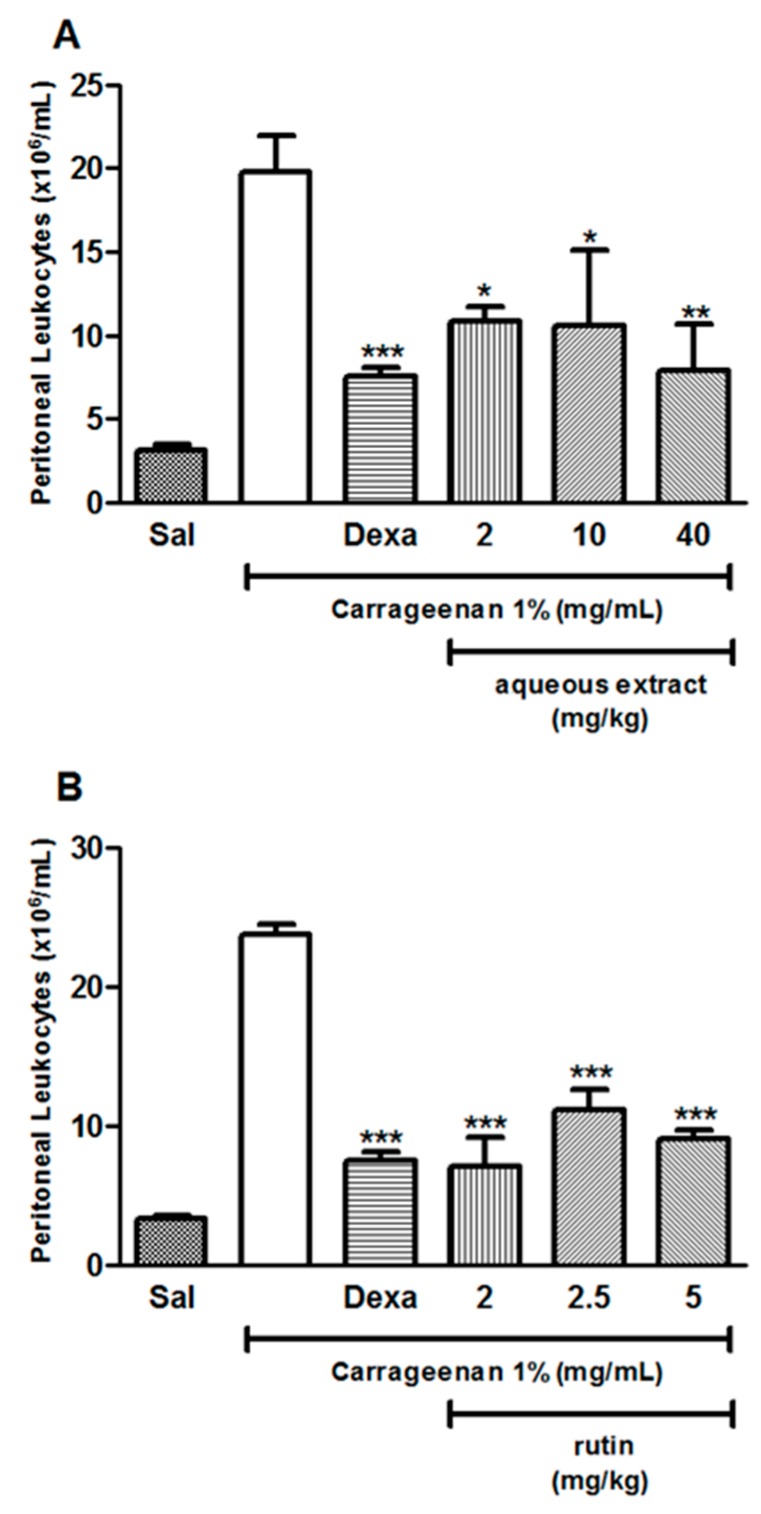
Effect of aqueous extracts from the leaves of *Aspidosperma pyrifolium* and rutin on inflammation in carrageenan-induced peritonitis model. BALB/c mice were treated intravenously with: aqueous extracts at doses of 2, 10 or 40 mg/kg (**A**); or rutin at doses of 2, 2.5 or 5 mg/mL (**B**), and immediately injected with 1 mL carrageenan 1% intraperitoneal. Dexamethasone (Dexa) at the dose of 0.5 mg/kg was used as positive control intraperitoneal. After four hours, the peritoneal wash was performed with PBS and the number of cells was determined using a Neubauer chamber. *** *p* < 0.001, ** *p* < 0.01 and * *p* < 0.05 when compared to the venom group. Data were analyzed using ANOVA followed by Tukey Test. Dexa: dexamethasone (0.5 mg/kg) and Sal: saline.

Aqueous extract of *A. pyrifolium* significantly inhibited the inflammation induced by carrageenan at 2, 10 and 40 mg/kg, showing a higher anti-inflammatory activity at a dose of 40 mg/kg (Figure 3A); rutin at doses of 2, 2.5 and 5 mg/kg also inhibited carrageenan-induced inflammation (Figure 3B). The effect of rutin on the cell count was equivalent to the effect of dexamethasone treatment 0.5 mg/kg. Table 1 summarizes the anti-inflammatory activity of aqueous extract and rutin in carrageenan-induced peritonitis model.

### 2.5. Effect of Dose and Time-Kinetics of Tityus serrulatus Venom-Induced Inflammation

**Figure 4:**
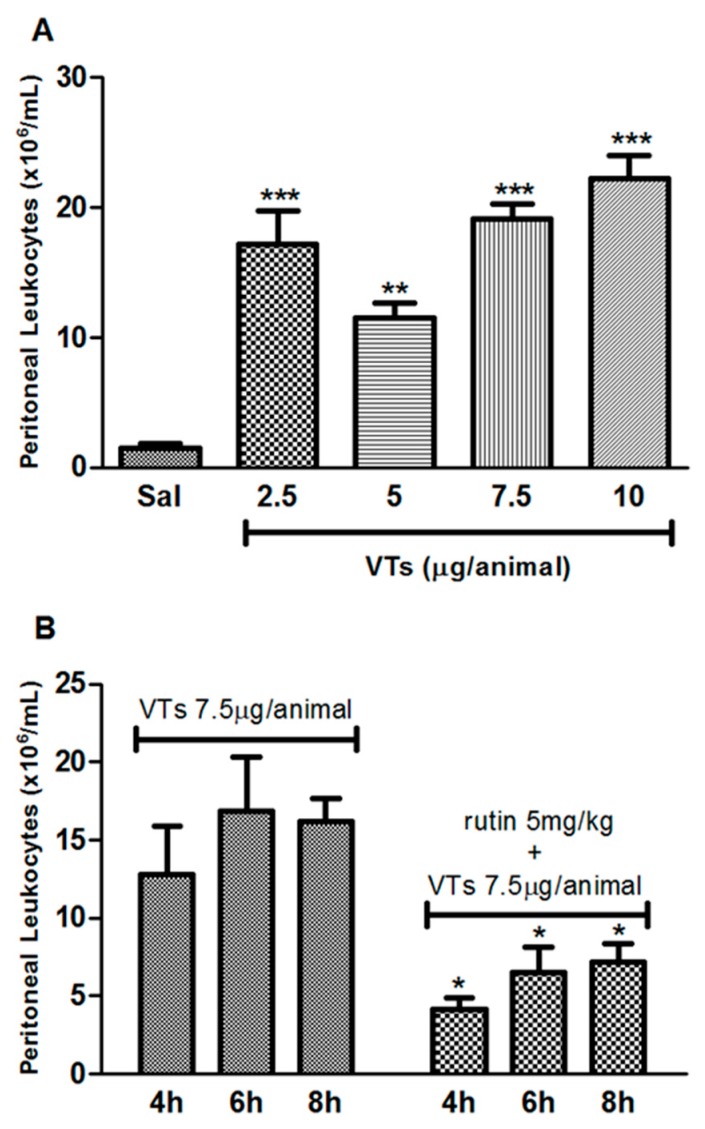
Effect of dose and time-kinetics of *Tityus serrulatus* venom-induced inflammation. Effect of venom dose on inflammatory response in envenomation-induced peritonitis model in mice. Animals received an intraperitoneal injection of 100 μL of VTs (2.5, 5, 7.5 and 10 μg/animal) and 100 μL (intravenous) of saline. After six hours, mice were euthanized and peritoneal wash and total cell counts were performed (**A**). Time-kinetics of VTs-induced inflammation and of rutin treatment on the envenomation-induced peritonitis model in mice. Animals received an intraperitoneal injection of 100 μL of VTs (7.5 μg/animal) and were treated with saline or rutin 5 mg/kg (intravenous). Peritoneal wash and total cell counts were performed at indicated time-points after envenomation (**B**). *** *p* < 0.001, ** *p* < 0.01 and * *p* < 0.05 when compared to the venom group. Data were analyzed using ANOVA followed by Tukey Test. Sal: saline and VTs: *Tityus serrulatus* venom.

Figure 4A shows that the dose of 7.5 μg/animal of VTs induced a higher leukocyte migration without causing death of the animals; therefore, it was selected for further envenomation experiments. In turn, Figure 4B reveals that the time of choice for euthanizing the animals was 6 h after envenomation, due to increased cell migration at that time-point. The time-kinetic results in Figure 4B also revealed that treatment with rutin at the dose of 5 mg/kg has an anti-inflammatory activity against inflammation induced by VTs at all tested time-points.

### 2.6. Evaluation of Rutin and Aqueous Extract of the Leaves of Aspidosperma pyrifolium in Tityus serrulatus Venom-Induced Peritonitis Model

**Figure 5:**
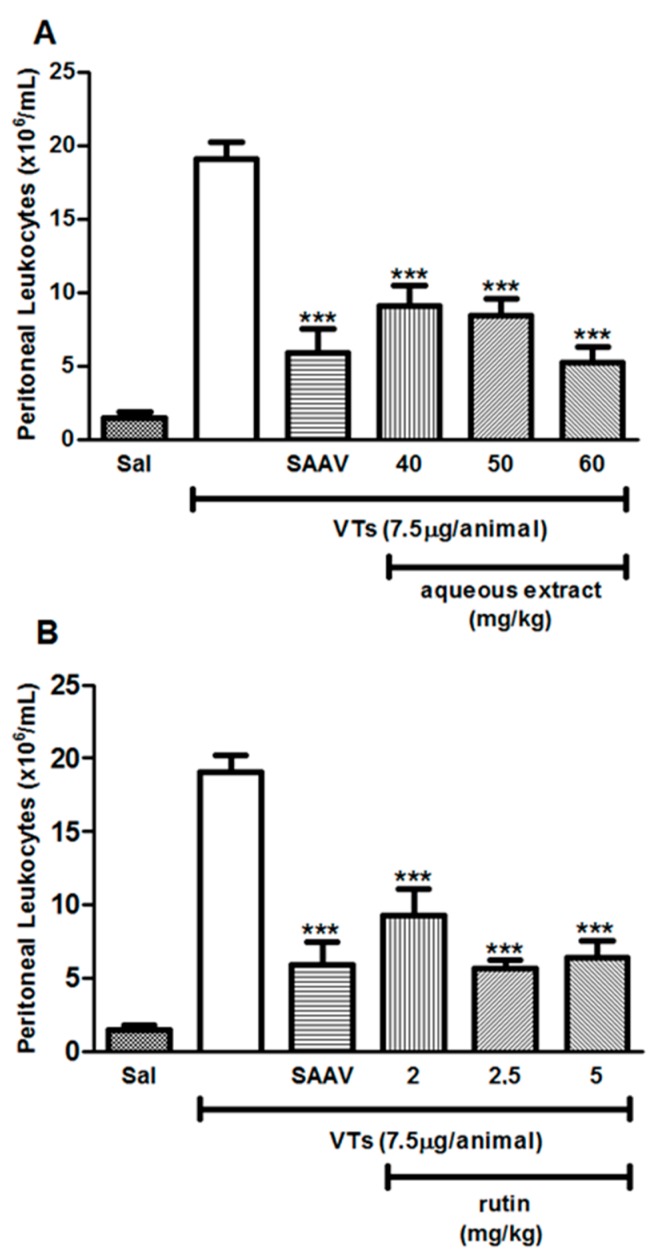
Anti-inflammatory effect of aqueous extract from the leaves of *Aspidosperma pyrifolium* and rutin on VTs-induced peritonitis model. The animals received an intraperitoneal injection of 100 μL of VTs and were treated at the same time with: AE (40, 50 and 60 mg/kg, intravenous) (**A**); or rutin (2, 2.5 and 5 mg/kg, i.v.) (**B**). Saline or arachnidic antivenom (SAAV, 50 μL/animal, intravenous) was used as negative or positive control, respectively. The peritoneal cell infiltration was analyzed after 6 h. *** *p* < 0.001 when compared to the venom group. Data were analyzed using ANOVA followed by Tukey Test. Sal: saline; SAAV: arachnidic antivenom and VTs: *Tityus serrulatus* venom.

Groups envenomated and treated (i.v.) simultaneously with aqueous extract of *A. pyrifolium* at 40, 50, and 60 mg/kg (Figure 5A), or rutin at 2, 2.5 and 5 mg/kg (Figure 5B), showed a significant reduction, at all doses, in the migration of cells into the peritoneal cavity, in the same way as the group treated with arachnidic antivenom (SAAV). The peritoneal cell infiltration was analyzed after 6 h. Table 2 summarizes the anti-inflammatory activity of rutin and aqueous extract and rutin in venom-induced peritonitis model.

### 2.7. Histopathology Analysis

**Figure 6:**
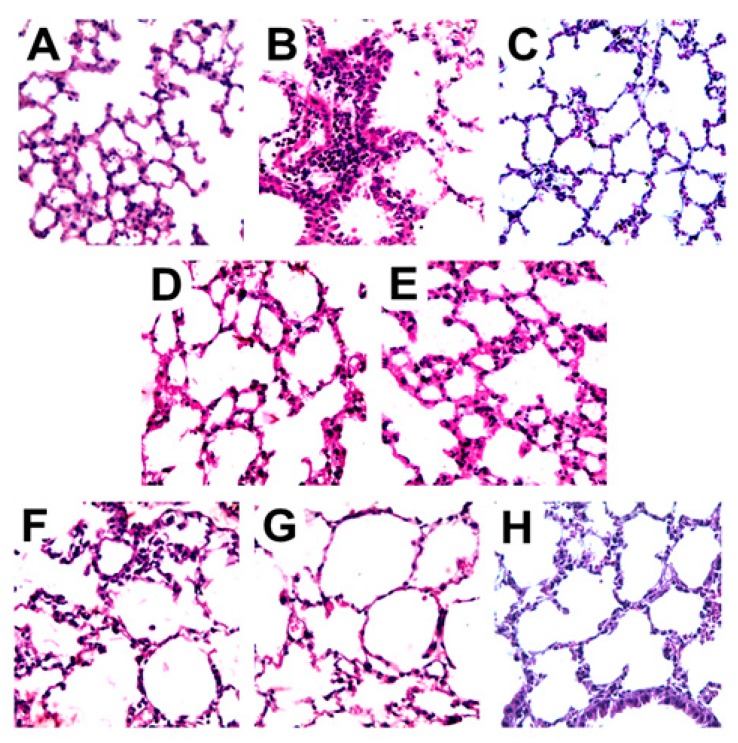
Effects of aqueous extract from the leaves of *Aspidosperma pyrifolium* and rutin in lung histopathologic changes in VTs-induced peritonitis model. The animals received 100 μL of VTs intraperitoneal and were treated intravenously, at the same time with saline, AE (40, 50 or 60 mg/kg) or rutin (2.5 or 5 mg/kg). Six hours after envenomation induction, the animals were euthanized and had their lungs removed, processed and analyzed by hematoxylin and eosin staining on optical microscope (40×): (**A**) saline (intraperitoneal) and saline (intravenous); (**B**) VTs (intraperitoneal) and saline (intravenous); and (**C**–**H**) animals treated (intravenous) with: (**C**) SAAV 50 μL; (**D**) rutin 2.5 mg/kg; (**E**) rutin 5 mg/kg (**F**) AE 40 mg/kg; (**G**) 50 mg/kg and (**H**) 60 mg/kg.

Edema and vascular ectasia were observed in the lung tissues of envenomed mice (Figure 6B). When compared with the saline group (Figure 6A), the lung tissues of envenomed mice presented a dense inflammation in the perivascular and peribronchiolar areas, and thickened interstitial space (alveoli walls) due to intense inflammation. In the group treated with serum arachnid antivenom, the number of inflammatory cells surrounding the bronchi and bronchioles and the alveolar wall thickness presented a similar histomorphological appearance as the saline group. Furthermore, vascular ectasia and edema were also similar between the control group and the SAAV-treated group (Figure 6C). The groups treated with rutin at the dose 2.5 or 5 mg/kg (Figure 6D,E, respectively) and aqueous extracts at the dose 40, 50 or 60 mg/kg (Figure 6F–H, respectively) of *A. pyrifolium* had a reduction in the number of inflammatory cells and the surrounding thickness of the alveolar wall; the groups treated with 50 mg/kg aqueous extracts of *A. pyrifolium* or 2.5 mg/kg rutin showed a closer similarity to the saline group.

## 3. Discussion

The present study was conducted to evaluate the protective ability of aqueous extract from the leaves of *Aspidosperma pyrifolium* against inflammation induced by carrageenan and *Tityus serrulatus* scorpion venom. Previous studies of chemical composition of *A. pyrifolium* reported the presence of alkaloids in the leaves and stem of this species [17,31]. However, in this approach, with the aid of HPLC-DAD and LC-DAD-MS analyses, the presence of flavonoids and phenolic acid were observed in aqueous extract of *A. pyrifolium* leaves. Rutin, one of the major compounds, and seven other phenolic derivatives, namely neo-chlorogenic acid, chlorogenic acid isomer (*n*-chlogenic, crypto-chlorogenic and iso-chlorogenic acid), isorhamnetin-3-*O*-rutinoside, di-*O*-caffeoylquinic acid and isomer of the di-*O*-caffeoylquinic acid, were identified by LC-DAD-MS. The presence of rutin was confirmed by comparison with reference standard data. These compounds are polyphenolic compounds that occur naturally in various plant species. The rutin show numerous interesting biological activities, for example, antioxidant capacity, anti-inflammatory action and stimulation of the immune system [23,24]. 

To evaluate the cell viability effect of aqueous extracts of *A. pyrifolium* in non-tumor cells (3T3), we have performed the 3-(4,5-dimethythiazol-2-yl)-2,5-diphenyl-2H-tetrazolium (MTT) assays. The data suggest that the doses of *A. pyrifolium* extracts used were safe, especially until 48 h. Further assays should be done to verify if the extract may present cytotoxic activity against tumor cells, as observed in *Himatanthus drasticus*, a medicinal plant from the same family, of which the uses in traditional medicine are supported by reports of its uses in cancer treatment, as an anti-inflammatory medication, and to stimulate the immune system [32].

Previous studies evaluated the anti-inflammatory activity of plants in carrageenan-induced peritonitis assay in murine model, with the aim of assessing the activity of isolated compounds [27,28]. This model is a well-characterized experimental model of acute inflammation, largely employed to evaluate new anti-inflammatory therapies focusing on quantify or analyze peritoneal vascular permeability and cellular migration as well as changes in other inflammatory parameters [33,34,35,36]. Carrageenan is a standard phlogistic agent, which induces inflammatory responses, such as infiltration of inflammatory cells, release of inflammatory mediators, increase in capillary permeability and peritonitis [37,38]. Leukocytes are involved in organism defense, and their migration into the tissue is a crucial process during host response against microorganism infections and injurious agents [33]. The effect of *A. pyrifolium* on acute inflammatory response in mice was evaluated four hours after carrageenan injection in the peritoneal cavity of the animals. The carrageenan-induced peritonitis resulted in an increase in total number of cells that migrated into the peritoneal cavity, and was observed the anti-inflammatory effect of aqueous extracts of *A. pyrifolium* (2, 10 and 40 mg/kg, i.v.) in the treatment of the animals, since total leukocyte migration to the site of inflammation significantly decreased. These findings corroborate previous studies demonstrating that anti-inflammatory effect is a common property of plants from the Apocynaceae family [17,32,39].

Envenomation by scorpion of genus *Tityus* spp. is more serious for children than for adults [1,3,4,5]. The toxicity of the venom can be related to the age of individuals and their body weight; children have lower body weight when compared to adults, and are thus more responsive to the envenomation [3,40]. Several mechanisms are involved in the systemic manifestations caused by the venom of *T. serrulatus*, including changes in the function of sodium, potassium and calcium ions in excitable cells, nitric oxide and cytokine receptors. These changes have been implicated in the severity of envenoming [15,16,40,41]. In this study, the effect of *A. pyrifolium* on acute inflammatory response in mice was evaluated six hours after VTs injection in the peritoneal cavity of the animals. The results showed that there was a reduction in the total number of cells that migrated into the peritoneal cavity in animals treated with aqueous extracts (40, 50 and 60 mg/kg, i.v.). These results are in agreement with previous studies, which assessed the anti-inflammatory activity of plants from Apocynaceae family against *T. serrulatus* venom [15,16]. 

Here, we also evaluated the effect of treatment with the component rutin on acute inflammatory response in mice, four hours after carrageenan injection in the peritoneal cavity of the animals, as well as its anti-inflammatory effect on mice six hours after injection of VTs in the peritoneal cavity. The main results confirmed its anti-inflammatory effect in two models of peritonitis at all doses tested (2, 2.5, 5 mg/kg, i.v.). Rutin is considered a potent antioxidant with a wide range of applications and has been a subject of interest due to its various pharmacological activities, in particular its anti-inflammatory activity, as well as improvements in strength and permeability of lymphatic and venous vessels walls [24,25,26,42]. In our study, we observed that rutin is a one of the components of AE involved in pharmacological effect observed in vivo models tested.

The venom of the scorpion *T. serrulatus* induces pulmonary edema resulting in an increase in lung permeability by the release of vasoactive substances. The pathogenesis of such edema is very complex; envenoming by this species can be severe, and lead to death caused by acute pulmonary edema [5,6]. In the model used in this study, histological analyses of the lung were well in agreement with peritoneal cell counts results, with a significant reduction of inflammation induced by VTs, and inhibition of the migration of inflammatory cells to the lungs by *A. pyrifolium* and rutin. This is the first study focusing on the ability of rutin and aqueous extracts of *A. pyrifolium* to inhibit lung inflammation in a model of venom-induced peritonitis. It was found that the treatment with rutin and aqueous extracts decreased the cell inflammation and yielded histological findings similar to the control group and the SAAV-treated group, corroborating the results of peritoneal infiltration shown in the present study.

## 4. Materials and Methods

### 4.1. Plant Material

The leaves of *Aspidosperma pyrifolium* were collected in the city of Mossoró, Rio Grande do Norte, Brazil (lat: −5.1875 long: −37.3442 WGS84), in December 2011. A voucher specimen (registration number MOSS 10481; 12 August 2008) was deposited in the “Dárdano Andrade Lima” Herbarium of “Universidade Federal Rural do Semi-Árido—UFERSA”. The botanical identification of the material was performed by Maíra Conceição Jeronimo de Souza Lima. The leaves were washed, dried at room temperature, and milled. The plant was collected under authorization of the Brazilian Access Authorization and Dispatch Component of Genetic Patrimony (CGEN) (Process number 010844/2013-9; 25 October 2013) and Brazilian Authorization and Biodiversity Information System (SISBIO) (process number 35017; 17 October 2017).

### 4.2. Extraction

The dried and ground leaves, which were left in contact with boiling water for 15 min, were extracted by decoction in a 1:10, proportion of plant:solvent (*w*/*v*). Then, the aqueous extract was filtered, frozen (−80 °C) and freeze-dried (Liobras^®^, model L 202, São Paulo, Brazil) to provide the dried aqueous extract (AE) for performing in vivo and analytical studies.

### 4.3. Qualitative High-Performance Liquid Chromatography Coupled with Diode Array (HPLC-DAD) Analysis

The analyses of AE *A. pyrifolium* was performed in a Varian Pro Star High Performance Liquid Chromatography equipped with a diode array detector (HPLC-DAD). The data were generated using the Galaxie™ software (Varian Inc, Palo Alto, CA, USA). Chromatographic analyzes were performed using a reversed phase C-18 column (Phenomenex^®^, 4.6 mm × 250 mm, 5 μm, Torrance, CA, USA). An elution system consisted of acetonitrile 100% (solvent A) and acetic acid 0.3% pH 3.0 (solvent B) at the following gradient flow was used: 0–5 min, an isocratic elution with A:B 7:93 (*v*/*v*); 5–17 min, a linear change from A:B 7:93 (*v*/*v*) to A:B 10:90 (*v*/*v*); 17–25 min, an isocratic elution with A:B 10:90 (*v*/*v*); 25–40 min, a linear change from A:B 10:90 (*v*/*v*) to A:B 23:77 (*v*/*v*); 40–50 min, an isocratic elution with A:B 23:77 (*v*/*v*). The flow rate was kept constant at 1.0 mL min^−1^, and the chromatograms were recorded at 340 nm, whereas the UV spectra were monitored at wavelengths from 200 to 400 nm. Peaks were characterized by comparison of retention times and UV spectra with reference standards and by co-injection (aqueous extract + reference standard). The reference standard solution and AE from *A. pyrifolium* was prepared in methanol:H_2_O (3:2, *v*/*v*) at a concentration of 50 μg/mL and 3 mg/mL, respectively. All solutions prepared for HPLC analysis were filtered through a 0.45 μm nylon membrane before use. The AE was analyzed in triplicate. The rutin (purity 94%) was purchased from Sigma-Aldrich^®^, St. Louis, MO, USA.

### 4.4. Liquid Chromatography Coupled with Mass Spectrometry with Diode Array Detection Analysis (LC-DAD-MS) Analyses

The analyses by LC-MS were performed using a Shimadzu LC-20AD equipment with a micrOTOFII (Bruker Daltonics, Billerica, MA, USA) ESI-qTOF mass spectrometer in high resolution and the same LC equipment coupled to amaZon (Bruker Daltonics) ESI-ion trap mass spectrometer generated a Low resolution spectrum. The column (Luna 5 μ C18, 250 × 4.6 mm, Phenomenex^®^), connected in line, were used for chromatographic analyses. The flow rate was 1.0 mL min^−1^; injection volume of 20 μL; mobile phase was acetonitrile (B) and H_2_O (A); added acetic acid 0.3% (*v*/*v*); and the elution profile: 13% B in 0–5 min, 13–18% B in 5–40 min, 18–19% B in 40–45 min, 19–23% B in 45–55 min, 23–13% B in 55–57 min, and 13% B in 57–58 min. It was used a split with the column eluent at a ratio of 7:3, going to the DAD detector and to the mass spectrometer, respectively. The range of mass were recorded between *m*/*z* 100 and 1300 in positive and negative modes, the spectrum was obtained in high resolution, using capillary and end plate with 3.5 KV and 500 V, respectively. The gas nebulizer used was nitrogen with a rate flow of 10 L min^−1^ and drying gas temperature of 220 °C. The solvents used were HPLC-grade and ultra-pure water.

### 4.5. Animals

Male and female BALB/c mice (25–35 g), 6–8 weeks of age, were received from “Animal Facility of the Center for Health Sciences” at Federal University of Rio Grande do Norte (UFRN) and maintained under controlled temperature (22 ± 2 °C), light/dark cycle of 12 h and with free water and commercial feed. Five animals were used in each group. After the experiments, all animals were euthanized with an overdose of sodium thiopental (>100 mg/kg, intraperitoneally). All experiments were approved by the Committee for Ethics in Animal Experimentation of the UFRN (protocol No.: 031/2011; 7 December 2011) in accordance with ethical principles in animal research adopted by the Brazilian Society of Animal Science and the National Brazilian Legislation No. 11.794/08, 8 October 2008.

### 4.6. Venom and Antivenom

Lyophilized *T. serrulatus* scorpion venom (VTs) was kindly supplied by the Butantan Institute, São Paulo. The venom was prepared in physiological saline at 1 mg/mL and stored at −20 °C until used. The arachnidic antivenom (SAAV, batch No. 1112256) was derived from plasma of hyperimmunized horses with venoms from the spiders *Loxosceles gaucho* and *Phoneutria nigriventer* and venom from the scorpion *T. serrulatus* (5 mL/vial; 1 mL neutralizes 1 mg of *T. serrulatus* reference venom in mice), Butantan Institute, São Paulo, Brazil. The use of *T. serrulatus* scorpion venom was developed under authorization of “Brazilian Access Authorization and Dispatch Component of Genetic Patrimony (CGEN)” (Process 010844/2013-9, 25 October 2013).

### 4.7. Cell Proliferation Assay

The effect of AE of *A. pyrifolium* in fibroblasts cells (3T3, CRL-1658) was determined using the colorimetric assay MTT 3-(4,5-dimethythiazol-2-yl)-2,5-diphenyl-2H-tetrazolium as described by Almeida-Lima, et al. (2010) [43]. Briefly, the cells were seeded in 96 well plates at a density of 5 × 10^3^ cells in 200 μL of DMEM (10% FBS) per well, and allowed to attach overnight at 37 °C and 5% CO_2_. Then, the medium was replaced by serum-free medium for synchronizing the cell cycle in G0 phase. After 12 h, the medium was replaced by the aqueous extract at concentrations ranging from 0.25 to 1.75 mg/mL in DMEM (10% FBS), and incubated for 48 h or 72 h. After each time-point, the sample-containing medium was removed and MTT, 1 mg/mL in serum-free medium, was added. After 4 h, MTT was removed, ethanol P.A. was added to solubilize formazan crystals, and the absorbance was measured with a spectrophotometer plate reader at 570 nm. The experiment was performed in triplicate.

### 4.8. Carrageenan-Induced Peritonitis Model

Animals were randomly allocated in 9 groups (5 animals in each group), and treated with 100 μL, intraperitoneally (i.p.) or intravenously (i.v.): saline solution (Groups 1 and 2, i.v.); 0.5 mg/kg dexamethasone (Group 3, i.p.); 2, 10, or 40 mg/kg AE of *A. pyrifolium* (Groups 4, 5 and 6, respectively, i.v.); or 2, 2.5 or 5 mg/kg rutin (Groups 7, 8, or 9, respectively, i.v.). Simultaneously, the animals were injected (i.p.) with saline (group 1) or carrageenan (1 mg/mL, Groups 2–9). Carrageenan-induced peritonitis assay was performed according to the procedure described by Longhi-Balbinot et al. (2012) [33], with few modifications. Briefly, mice were anesthetized and pretreated as described above. Thirty minutes later, the animals received 1 mL of carrageenan 1% i.p. diluted in sterile saline. Four hours after peritonitis induction, mice were euthanized; 2 mL of cold sterile saline were injected in the peritoneal cavity and, after 30 s of gentle massaging, peritoneal fluid was collected. Exudates were centrifuged at 250× *g* for 10 min, at 4 °C, and the total cell counts were performed with a Neubauer chamber via optical microscopy, after diluting a sample of the peritoneal fluid in Turk’s solution.

### 4.9. Dose and Time-Kinetics of Tityus Serrulatus Venom-Induced Inflammation

Envenomation was first induced in various doses to verify the dose, which could cause an increased inflammation without promoting death of the animals. Envenomation was performed according to the procedure described by Pessini et al. (2003) [44], with few modifications. Animals were randomly allocated in five groups (5 animals in each group) and received 100 μL: Saline solution (Groups 1 and 2, i.v.); simultaneously, the animals were injected (i.p.) with saline (group 1) or VTs 2.5, 5, 7.5 and 10 μg/animal (Groups 2–5), respectively. After six hours of envenomation induction, the mice were euthanized; 2 mL of cold sterile saline were injected in the peritoneal cavity and, after 30 s of gentle massaging, peritoneal fluid was collected. Then, the exudates were centrifuged and total cell counts were performed. To verify the time with the highest cell migration for subsequent assays, the animals received an i.p. injection of 100 μL of VTs (7.5 μg/animal), and were euthanized after 4, 6 or 8 h for collecting and counting of peritoneal leukocytes.

### 4.10. Venom-Induced Peritonitis Model

To verify the activity of rutin against inflammation caused by VTs over time, animals received 100 μL of rutin 5 mg/kg (i.v.) and simultaneously, VTs (7.5 μg/animal). After 4, 6 or 8 h, mice were euthanized and the counting of peritoneal leukocytes was performed. 

After defining the dose and time for envenomation assays, the animals were randomly allocated into nine groups (5 animals in each group), and received 100 μL: Saline solution (Groups 1 and 2, i.v.); arachnid antivenom (SAAV) (Group 3, i.v.); 40, 50 and 60 mg/kg AE of *A. pyrifolium* (Groups 4, 5 and 6, respectively, i.v.); or 2, 2.5 and 5 mg/kg of rutin (Groups 7, 8 and 9, respectively, i.v.). Simultaneously, the mice received an i.p. injection of 100 μL of VTs (Groups 2–9). Six hours after envenomation induction, the animals were anesthetized and euthanized and the peritoneal leukocytes were counted to verify the activity of *A. pyrifolium* against inflammation caused by envenomation.

### 4.11. Histological Analysis

Lungs were fixed in 10% formalin for 24 h, paraffin embedded, processed according to the Laboratory of Pathology/UFRN protocol, sectioned at 5 μm and stained with hematoxylin and eosin to visualize the lung structure and inflammatory cell infiltration. These samples were coded and read by a blinded investigator using optical microscope Olympus C40× and photographed with an Olympus DP20 camera. The following parameters were assessed for the histopathological analyses: inflammatory cell infiltration and increased alveolar wall thickness. Inflammatory cell infiltration, i.e., the number of inflammatory cells present, was evaluated in the perivascular zone, the interstitial space (alveoli walls), in the bronchiolar epithelium and peri-bronchial alveolar tissue of each group.

### 4.12. Statistical Analyses

Data were expressed as mean ± standard deviation. Statistical analyses were performed by One-way ANOVA test with Tukey’s test were performed using GraphPad Prism version 5.00 (San Diego, CA, USA). The difference in the mean values of *** *p* < 0.001, ** *p* < 0.01 and *p < 0.05 were considered as statistically significant.

## 5. Conclusions

In conclusion, this approach demonstrated that aqueous extracts of *A. pyrifolium* can counteract the inflammatory effects induced by *T. serrulatus* scorpion venom in mice. The data also confirm the presence of the flavonoid and phenolic acids in the plant. Finally, we revealed that the lungs of animals treated with rutin and aqueous extracts of *A. pyrifolium* showed a reduction in inflammation in the dense perivascular and peribronchial areas, as well as the interstitial space (the walls of the alveoli). These results exhibited the anti-inflammatory action of the aqueous extract of *A. pyrifolium* and its bioactive molecules, especially rutin, at least in part, responsible for such activity. Further studies are required to test the potential application of this extract and identified compound in the therapy of inflammatory conditions and of scorpion envenomation.

## Figures and Tables

**Table 1:** Anti-inflammatory activity of aqueous extract (AE) from the leaves of *Aspidosperma pyrifolium* and rutin against carrageenan-induced peritonitis model.

Groups		Dose (mg/kg)	Peritoneal Cell Migration (×10^6^ cells/mL)	Inhibition (%)
Carrageenan 1%	saline	-	19.80 ± 2.189	
	dexa	0.5	7.500 ± 0.5701 ***	62.1
	AE	2	10.80 ± 0.9028 *	45.4
	AE	10	10.60 ± 4.529 *	46.4
	AE	40	7.900 ± 2.731 **	60.1
Carrageenan 1%	saline	-	23.70 ± 0.752	
	dexa	0.5	7.500 ± 0.5701 ***	62.1
	rutin	2	7.000 ± 2.133 ***	70.4
	rutin	2.5	11.10 ± 1.470 ***	53.1
	rutin	5	9.000 ± 0.6892 ***	62

Values are mean ± standard deviation (S.D.), *n* = 5, *** *p* < 0.001, ** *p* < 0.01 and * *p* < 0.05, compared to carrageenan group. Dexa: dexamethasone 0.5 mg/kg.

**Table 2:** Anti-inflammatory activity of aqueous extract (AE) from the leaves of *Aspidosperma pyrifolium* and rutin against *Tityus serrulatus* venom-induced peritonitis model.

Groups		Dose (mg/kg)	Peritoneal Cell Migration (×10^6^ cells/mL)	Inhibition (%)
*T. serrulatus* venom (7.5 μg/animal)	saline	-	19.10 ± 1.145	
	SAAV	-	5.875 ± 1.638 ***	69.6
	AE	40	9.083 ± 1.393 ***	52.8
	AE	50	8.417 ± 1.207 ***	56.0
	AE	60	5.250 ± 1.055 ***	72.7
*T. serrulatus* venom (7.5 μg/animal)	saline	-	19.10 ± 1.145	
	SAAV	-	5.875 ± 1.638 ***	69.6
	rutin	2	9.300 ± 1.758 ***	51.3
	rutin	2.5	5.700 ± 0.5612 ***	70.1
	rutin	5	6.400 ± 1.198 ***	66.4

Values are mean ± standard deviation (S.D.), *n* = 5, *** *p* < 0.001, compared to venom group. SAAV: Serum Arachnid antivenom (1 mg/mL).
